# Mortality trends in extremely premature neonates: insights from the CDC WONDER database from 1999 to 2023

**DOI:** 10.3389/fped.2025.1683346

**Published:** 2025-11-20

**Authors:** Rebecca Hammond, Martin Phan, Olivia Foley, Abubakar Tauseef

**Affiliations:** 1 Department of Medical Education, School of Medicine, Creighton University, Omaha, NE, United States; 2 Department of Internal Medicine, Creighton University School of Medicine, Omaha, NE, United States

**Keywords:** prematurity, neonate, disparity, mortality, demographic trends, United States

## Abstract

**Introduction:**

Premature birth is associated with significant morbidity and mortality. Risk increases with younger gestational age. The ICD-10-CM code classifies extremely immature neonates as being born at less than 28 weeks gestation. Antenatal and neonatal interventions have improved outcomes overall. This study evaluates trends in extreme prematurity related mortality to determine if outcomes are evenly distributed among demographic groups, including pre and post COVID data.

**Methods:**

The CDC WONDER database was utilized to gather data on extreme immaturity related mortality in infants <1 year old from 1999 to 2023. Joinpoint regression was subsequently utilized for data analysis, analyzing crude mortality rate (CMR), annual percent change (APC), and average annual percent change (AAPC), stratifying data by sex, race, region, and urban vs. rural locality.

**Results:**

Extreme immaturity resulted in 92,917 deaths among neonates in their first year of life from 1999 to 2023. Overall CMR significantly decreased across the study period [AAPC −1.14* 95% CI (−1.45, −0.83)], with both female and male neonates experiencing a significant decrease in CMR [AAPC −1.32* 95% CI [−1.63, −1.06] and AAPC −1.10* 95% CI [−1.57, −0.68] respectively]. Black or African American neonates had a higher CMR than all other racial and ethnic groups. Of all the racial and ethnic groups, CMR significantly decreased only for Black or African American and White neonates [AAPC −1.98* 95% CI [−2.62, −1.46] and AAPC −1.12* 95% CI [−1.42, −0.82] respectively]. All US regions experienced significant declines in CMR except for the West [AAPC −0.72 95% CI (−1.32, 0.19)]. CMR decreased in urban localities but did not decrease in rural localities [AAPC −0.93* 95% CI [−1.24, −0.59] vs. AAPC 0.12 95% CI [−0.51, 0.96]].

**Conclusions:**

While medical advancements have improved outcomes for neonates born extremely premature, these outcomes are not evenly distributed amongst demographic groups in the United States. There was no large change in trends associated with the COVID-19 pandemic.

## Introduction

Premature birth is a major cause of morbidity and mortality among neonates. Major causes of death in premature infants include pulmonary complications, including bronchopulmonary dysplasia and neonatal respiratory distress syndrome, infection, central nervous system (CNS) injury, and gastrointestinal (GI) complications (1). Rates of injury and death increase with lower gestational age (GA), with one recent prospective observational study of 10,877 infants identifying survival to discharge to be 10.9% in infants born at 22 weeks as compared to 94.0% in infants born at 28 weeks (2). This trend is consistent across studies (3). The peri viable period is the GA range where survival outside of the uterus is relatively low and associated with major long-term complications. The American College of Obstetrics and Gynecology (ACOG) defines the peri viable period as 20 0/7 weeks to 25 6/7 weeks (3). The limit of viability exists between 22 and 24 weeks secondary to critical fetal lung development at that stage (4). Over the last three decades, there has been increasing resuscitation and survival of extremely premature neonates born within this range of limit of viability (3). While in the mid 1960s mortality was 95% for infants born at less than 1,000 g, by the 2000s survival was 95% (5).

Outcomes have improved for premature infants with advances in obstetric and neonatal care practices (2, 6, 7). Such advances in care practices include the use of antenatal interventions including corticosteroids, tocolysis, antibiotics, magnesium, and site and mode of delivery along with neonatal interventions. In the 1990s, many changes occurred in the care of premature infants. Consensus on the use of antenatal corticosteroid administration was published in the 1990s and surfactant was first approved in this decade as well (5). Nasal CPAP first began in the 1990s as well, with high flow nasal cannula appearing in the 2000s (8). Since this time, interventions have only continued to develop. While antenatal and neonatal practices have improved neonatal outcomes, it is unknown if these outcomes are evenly distributed amongst all demographic groups of extremely premature neonates. Previous studies have identified racial disparities in neonatal mortality amongst very low birth weight infants in the 1990s and early 2000s (9). Additionally, despite improved antenatal care, preterm births are increasing, with a greater than 10% increase from 2014 to 2022 in the United States (US) (10). Thus, it is important to continue to evaluate trends in mortality in premature infants given the rise in preterm birth.

This paper sought to investigate trends as well as disparities in neonatal mortality from 1999 to 2023 in infants born at a GA less than 28 weeks, as neonates at these ages experience most of the morbidity and mortality of all preterm neonates. Given the rise of interventions and improvement in mortality in the 1990s, the study period began in 1999, ending in 2023 to include years following the COVID-19 pandemic. While disparities in infant mortality have been established in the literature, trends in disparities have not been well documented in extremely premature infants. This study seeks to specifically identify trends in mortality and associated disparities in this population of infants. Additionally, this study includes data post-COVID to evaluate the impact of the COVID-19 pandemic on neonatal mortality in extremely premature infants.

## Materials and methods

The Centers for Disease Control and Prevention Wide-ranging Online Database for Epidemiologic Research (CDC WONDER) database was used to assess mortality related to extreme immaturity of the newborn in the US from 1999 to 2023. The mortality data is derived from death certificates of US residents. This database has been utilized in numerous studies to analyze mortality trends for specific conditions. In this study, we utilized the International Classification of Diseases, 10th Revision, Clinical Modification (ICD-10-CM) code P07.2 for extreme immaturity of the newborn which includes neonates born at less than 28 weeks of gestation (11).

The CDC WONDER database was used to stratify mortality data by various demographic variables, including sex, race/ethnicity, census regions, and urban-rural location. Racial and ethnicity groups were defined as non-Hispanic (NH) White, NH Black, NH American Indian/Alaskan Native, NH Asian/Pacific Islander, and Hispanic. Urban-rural classification is determined by the National Center for Health Statistics Urban-Rural Classification Scheme (12). The large central metro category is the most “urban” category, and the noncore category is the most “rural” category. The large central metro category contains counties in metropolitan statistical areas (MSAs) of more than one million, the large fringe category contains remaining counties of one million or more, counties in MSAs of 250,000–999,999 are medium metro, counties with MSAs under 250,000 are the small metro category, non-metropolitan counties were assigned to the noncore category (12). CDC WONDER data only includes rural vs. urban classification up to the year 2020, so data from 2021 to 2023 was not stratified by this specific demographic variable. Regions were classified into Northeast, Midwest, South, and West according to the Census Bureau and Health and Human Services definitions (13).

Crude mortality for each demographic group was calculated. The Joinpoint Regression Program (Joinpoint version 4.9.0.0 available from National Cancer Institute, Bethesda, Maryland) was used to determine trends in mortality from 1999 to 2023 (14). This program utilizes best-fit models to highlight linear segments where trends are stable as well as points in time (Joinpoints) where significant changes in trends occurred. Utilizing this program allowed for calculation of annual percentage change (APC) across linear segments and average annual percent change (AAPC) across the study period with 95% confidence intervals (CIs) for corresponding CMRs. These were calculated via the line segments linking Joinpoints using the Monte Carlo permutation test (15). APC and AAPCs were considered to statistically significant if the slope describing the change in mortality over the time interval was significantly different from zero using 2-tailed *t* test. Statistical significance was set at *p* ≤ 0.05. Asterisks, ‘*’, were used to denote significance.

## Results

### Crude mortality rate overall

Overall, from 1999 to 2023, there were 92,917 deaths related to extreme immaturity of the newborn in the US. From 1999 to 2003, CMR significantly increased [APC 5.46* 95% CI (3.33,8.89), *p* < 0.000001]. From 2003 to 2016, CMR significantly decreased [APC −1.38* 95% CI (−1.79, −0.89), *p* = 0.0008]. From 2016 to 2023, CMR again significantly decreased [APC −4.30* 95% CI (−6.00, −3.35), *p* < 0.000001]. Overall, during the study period, CMR significantly decreased with an AAPC of −1.14* [95% CI (−1.45, −0.83), *p* < 0.000001]. Joinpoint graphs are included in the Supplementary Material along with yearly CMRs.

### Crude mortality rate by sex

**Figure 1:**
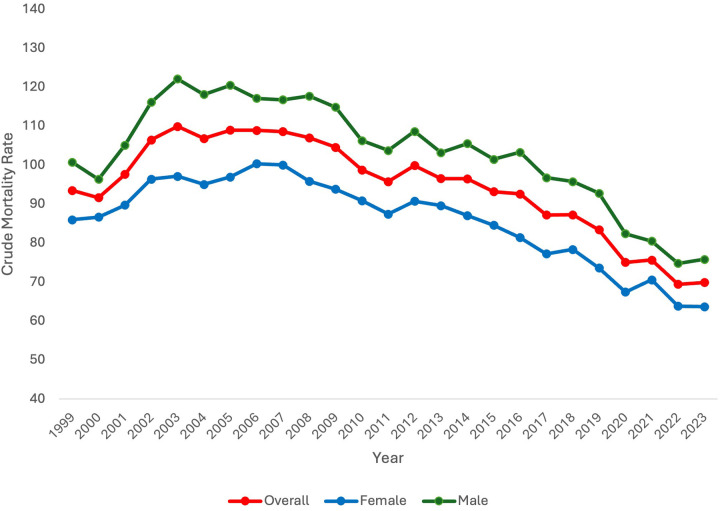
Extreme prematurity related mortality trends overall and stratified by sex, 1999–2023.

When stratifying by sex, CMR differed between males and females. Specifically, CMR was consistently higher in males in comparison to females. Like overall CMR, CMR in females and males increased at the beginning of the study period. In females, CMR significantly increased from 1999 to 2006 [APC 2.05* 95% CI (0.96,3.76), *p* = 0.027]. In males, CMR significantly increased 1999–2003 [APC 6.31* 95% CI (3.68, 11.54), *p* = 0.0004]. Subsequently, CMR did not significantly change for females from 2006 to 2015 but did significantly decrease after 2015 until the end of the study period [APC −3.58* 95% CI (−6.14, 2.75), *p* < 0.000001]. In males, CMR significantly decreased from 2003 to 2017 [APC −1.47* 95% CI (−1.95, −0.92), *p* = 0.0016] and from 2017 to 2023 [APC −4.93* 95% CI (−8.27, −3.56), *p* < 0.000001]. Overall, from 1999 to 2023, CMR significantly decreased in both females and males [AAPC −1.32* 95% CI [−1.63, −1.06], *p* < 0.000001 and AAPC −1.10* 95% CI [−1.57, −0.68], *p* < 0.000001, respectively] (Figure 1).

### Crude mortality rate by race and ethnicity

**Figure 2:**
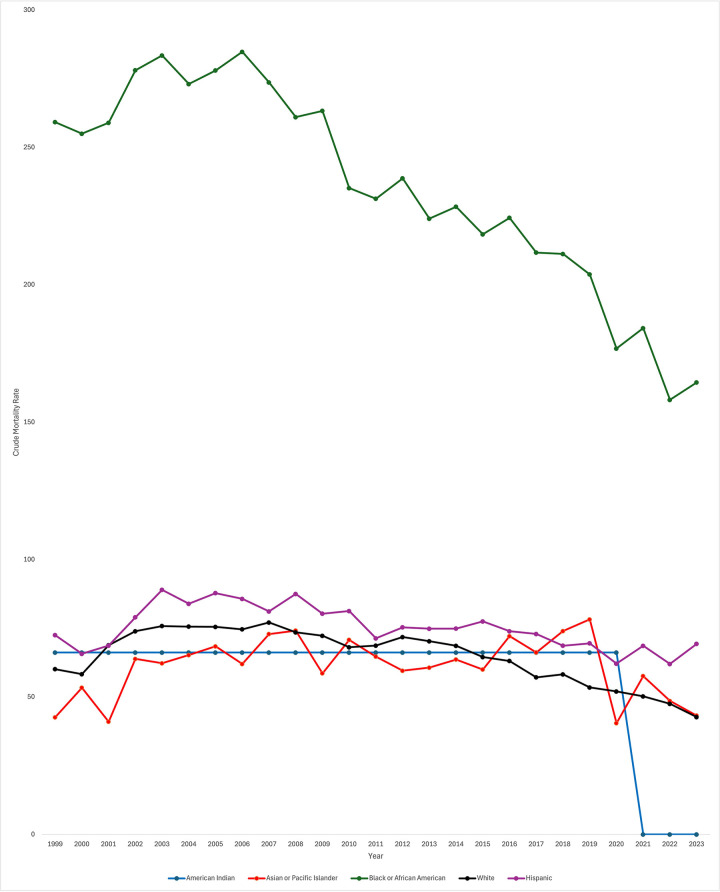
Extreme prematurity related mortality trends stratified by race and ethnicity, 1999–2023.

Data was also stratified by race including White, Black or African American, Asian or Pacific Islander, Hispanic, and American Indian populations. In White patients, CMR significantly decreased throughout the study period [AAPC −1.12* 95% CI (−1.42, −0.82), *p* < 0.000001]. CMR did similarly decrease in Black or African American patients [AAPC −1.98* 95% CI (−2.62, −1.46), *p* < 0.000001]. However, throughout the study period, CMR was consistently higher in Black or African American patients in comparison to all other races. CMR did not significantly change over the study period for Asian or Pacific Islander and Hispanic patients [AAPC −1.36 95% CI [−2.99, 0.051], *p* = 0.058 and AAPC 0.13 95% CI [−0.57, 0.42], *p* = .57, respectively]. In American Indian patients, AAPC was 0* from 1999 to 2020 (95% CI, *p* = 0.0004) (Figure 2). Data from 2021 to 2023 was unreliable in American Indian patients and thus not used in the analysis.

When looking specifically at White patients, CMR significantly increased from 1999 to 2003 [APC 7.25* 95% CI (4.98, 10.76), *p* < 0.000001]. From 2003 to 2014, CMR significantly decreased [APC −1.09* 95% CI (−1.65, −0.44), *p* = 0.007199] and again from 2014 to 2023 [APC −4.66* 95% CI (−5.79, −3.92), *p* < 0.000001]. Again, overall CMR significantly decreased throughout the study period for White patients [AAPC −1.12* 95% CI (−1.42, −0.82), *p* < 0.000001].

When looking specifically at Black or African American patients, discrete data trends were identified from 1999 to 2005 and 2005–2018 but were not statistically significant [APC 1.55 95% CI [−2.57, 6.93], *p* = 0.13 and APC −2.38 95% CI [−2.99, 5.12], *p* = 0.12, respectively]. From 2018 to 2023, CMR did significantly decrease (APC −5.05* 95% CI [−11.91, −2.77], *p* = 0.002. As noted previously, overall CMR did significantly decrease across the entire study period.

CMR in Asian or Pacific Islander patients significantly increased throughout the majority of the study period, from 1999 to 2019 [APC 1.03* 95% CI (.059, 2.89), *p* = 0.040]. During the last four years of the study period, CMR significantly decreased [APC −12.49* 95% CI (−32.10, −3.31), *p* = 0.0012]. Again, overall, CMR did not significantly change across the study period.

CMR did not significantly change in American Indian patients. Discrete trends were identified in the data, though APC was similarly 0 for both time periods. From 1999 to 2002, APC was 0 (95% CI 0, *p* = 0.34). From 2002 to 2020, APC again was 0.00* (95% CI 0, *p* = 0.0056). Data was unreliable for American Indian patients from 2021 to 2023; therefore, data was only trended through the year 2020. As noted previously, the overall trend from 1999 to 2020 was no significant increase or decrease in CMR (AAPC 0.00* 95% CI, *p* = 0.0004).

When looking specifically at Hispanic patients, CMR significantly increased from 1999 to 2004 [APC 5.57* 95% CI (2.44, 11.55), *p* = 0.0004]. From 2004 to 2023, CMR significantly decreased [APC −1.58* 95% CI (−2.09, −1.17), *p* < 0.000001]. As described previously, when analyzing the entire study period, CMR did not significantly change across the study period [AAPC 0.13 95% CI (−0.57, 0.42), *p* = .57].

### Crude mortality rate by region

**Figure 3:**
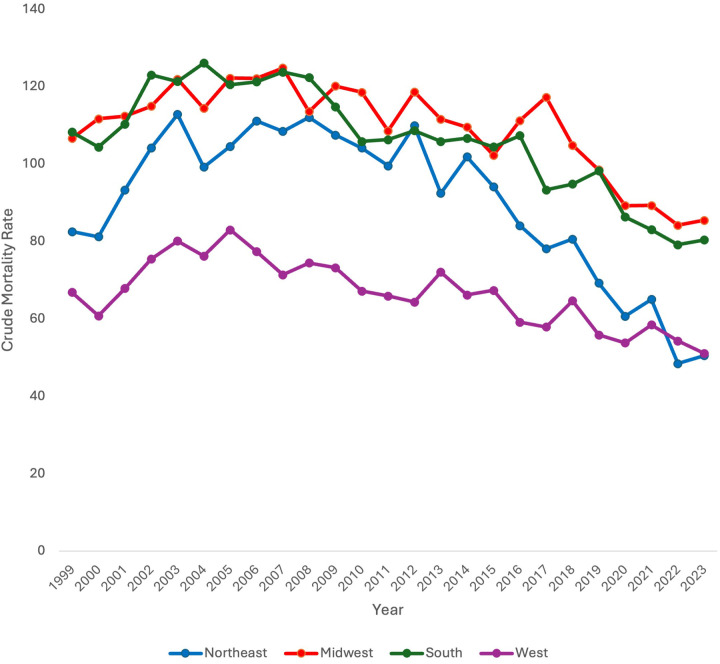
Extreme prematurity related mortality trends stratified by region, 1999–2023.

Data was stratified by census region across the US including Northeast, Midwest, South, and West. When stratifying by census region, CMR was found to significantly decrease in all census regions (Northeast, Midwest, and South) throughout the study period except for the West (AAPC −1.89* 95% CI [−2.50, −1.26), *p* < 0.000001; AAPC −1.17* 95% CI [−1.60, −0.77), *p* < 0.000001; AAPC −1.21* 95% CI [−1.71, −0.74], *p* < 0.000001 vs. AAPC −0.72 95% CI [−1.32, 0.19], *p* = 0.072, respectively) (Figure 3).

Discrete trends were also identified in each region. In the Northeast, CMR significantly increased from 1999 to 2003 (APC 8.40* 95% CI [4.38, 17.63, *p* < 0.000001). It did not significantly change from 2003 to 2014 [APC −0.73 95% CI (−1.97, 0.39), *p* = .18]. From 2014 to 2023, CMR did significantly decrease [APC −7.47* 95% CI (−9.70, −6.09), *p* < 0.000001].

In the Midwest, CMR significantly increased from 1999 to 2005 [APC 2.05* 95% CI (0.52,6.74), *p* = 0.016]. From 2005 to 2017, CMR significantly decreased [APC −0.98* 95% CI (−1.93, −0.21), *p* = 0.017]. CMR further significantly decreased from 2017 to 2023 [APC −4.65* 95% CI (−8.71, −3.10), *p* = 0.0076].

In the South, CMR significantly increased from 1999 to 2004 [APC 3.59* 95% CI (0.72, 9.17), *p* = 0.040]. It did not significantly change from 2004 to 2016 [APC −1.73 95% CI (−2.35, 3.76), *p* = .11] but did significantly decrease from 2016 to 2023 [APC −3.64* 95% CI (−9.37, −2.32), *p* = .0016].

In the West, CMR significantly increased from 1999 to 2004 [APC 4.90* 95% CI (1.12, 14.30), *p* = 0.014]. CMR subsequently significantly decreased from 2004 to 2023 [APC −2.15* 95% CI (−3.01, −1.64), *p* < 0.000001].

### Crude mortality rate by rural vs. urban locality

**Figure 4:**
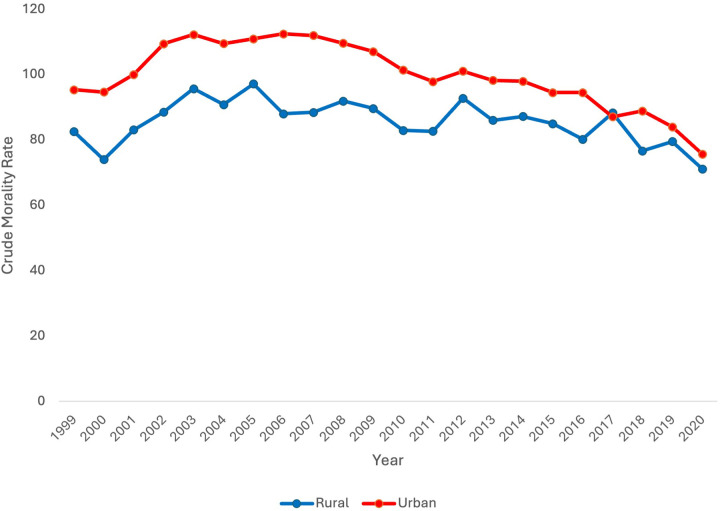
Extreme prematurity related mortality trends stratified by rural vs. urban locality, 1999–2020.

Finally, data was stratified by urban vs. rural locale. Urban and rural data stratification was only available between 1999 and 2020 on the CDC WONDER database. CMR was consistently higher in urban vs. rural locales except for the year 2017. From 1999 to 2020, CMR decreased only in the urban population with an AAPC of −0.93* [95% CI (−1.24, −0.59), *p* < 0.000001]. CMR did not decrease overall in the rural population [AAPC 0.12 95% CI (−0.51, 0.96), *p* = 0.72] (Figure 4).

In the rural population, CMR significantly increased from 1999 to 2003 [APC 5.31* 95% CI (1.00, 15.49), *p* = 0.016]. From 2003 to 2020, CMR significantly decreased [APC −1.06* 95% CI (−2.04, −0.56), *p* = 0.0008].

In the urban population, CMR significantly increased from 1999 to 2003 [APC 4.63* 95% CI (2.53, 8.22), *p* = 0.0004]. There was no significant change in CMR from 2003 to 2007 [APC −0.17 95% CI (−2.52, 3.35), *p* = 0.80]. CMR significantly decreased from 2007 to 2018 and again from 2018 to 2020 [APC −2.02* 95% CI [−2.55, −0.68], *p* = 0.019; APC −7.02* 95% CI [−9.90, −3.43], *p* < 0.000001 respectively].

## Discussion

### Overall

Throughout the study period, overall CMR due to extreme immaturity significantly decreased. This would be expected with advances in medical care. Interestingly, from 1999 to 2003, there was a significant increase in CMR before CMR began to decrease. This could be due to changing attitudes about resuscitating extremely premature neonates with increasing resuscitation of 23–24-week-old neonates occurring at this time (16). It is important to note that improved survival does not necessarily imply improved morbidity (6).

### Sex

Data stratified by sex aligns with other studies which have demonstrated higher mortality for male neonates in comparison to female neonates. In an Australian retrospective cohort study of male infants born after 28 weeks, male neonates had higher rates of neonatal morbidity and mortality as well as rates of preterm birth (17). In a retrospective cohort study of neonates born in California, mortality was similarly lower in female neonates vs. male neonates from 24 to 28 weeks gestation (18). The explanation for this consistent trend is likely multifactorial in nature. Overall, for both male and female neonates, CMR significantly decreased across the study period, though it did initially increase at the beginning of the study period for both groups, consistent with the overall trend.

### Race and ethnicity

While overall CMR did decrease from 1999 to 2023, there were disparities in neonatal mortality rates based on race and ethnicity. Throughout the study period, CMR was consistently higher in Black or African American patients in comparison to all other races, although it did significantly decrease over the study period. Of note, there were only significant decreases in mortality for Black or African American patients from 2018 to 2023. Importantly, CMR did not decrease across the study period for Hispanic patients or Asian or Pacific Islander patients. Of note, for American Indian neonates, the AAPC was 0 from 1999 to 2020 and data from 2021 to 2023 was unreliable and could not be used. CMR did significantly decrease in White patients. Racial and ethnic disparities in neonatal mortality have been previously identified in older data sets, with non-Hispanic (NH) Black very low birth weight (VLBW) infants experiencing increased mortality in the early 2000s as opposed to NH White VLBW infants (9). Using the National Inpatient Sample (NIS) database from 2012 to 2018, researchers have similarly found increased neonatal mortality in Black neonates in comparison to White neonates, though this was not limited to premature neonates (19).

There are multiple potential explanations for these racial and ethnic differences. First, disparities in preterm birth rates leads certain neonates to be at higher risk of morbidity and mortality due to prematurity. Recent data has demonstrated an increase in preterm birth rates in the US (10). While they demonstrated similar increases across White, Black, and Hispanic mothers, they did see that Black mothers had a higher overall percentage of deliveries that were preterm in comparison to the other groups. In a study of state-level preterm birth rates, researchers found that only the percent of NH Black women by state was a significant predictor of state-level preterm birth rates after controlling for other risk factors for preterm birth (20).

Disparities in antenatal treatment may also influence subsequent disparities in neonatal mortality. It has been shown that NH Black and Hispanic women are less likely to receive antenatal corticosteroid treatment in comparison to NH White women (21, 22). NH Black women have also been shown to be less likely to receive tocolysis in comparison to NH White women (23). It is well established that antenatal corticosteroid treatment reduces the risk of neonatal death as well as neonatal morbidity including respiratory distress syndrome as well as intraventricular hemorrhage (24). Thus, disparities in receipt of an antenatal corticosteroid course is concerning given overwhelming support of a course in women at risk of preterm birth. The benefit of tocolytics on neonatal mortality are less certain, and tocolytics are associated with increased side effects; however, tocolytics are probably effective in delaying preterm birth (25). Tocolysis generally is used to allow time for other interventions to take place. Of note, other studies have not identified any difference in administration of antenatal corticosteroid by race (26).

Unfortunately, disparities also exist in neonatal care by race and ethnicity. A systematic review of neonatal intensive care identified not only inter-NICU disparities, with worse outcomes in NICUs primarily serving minority populations, but intra-NICU disparities, with disparities in care for minority patients when compared to white neonates in the same NICU (27). Therefore, the cause of disparities in mortality amongst extremely premature neonates is multifactorial, relating to antepartum and neonatal factors.

### Region

Neonatal mortality data was also stratified by region. CMR was found to decrease in all census regions except for the West. One possible explanation for this finding is regional variance in preterm births. However, in a study of county level variation in preterm birth rates from 2007 to 2019, researchers found that counties in the Southeast had higher preterm birth rates than other regions in the US (28). This suggests that it is not only disparities in preterm birth rates by region that are contributing to region-level variation in neonatal mortality for extremely premature neonates. Other explanations for this difference in mortality include differential access to appropriate care. It is well known that access to specialized delivery and postnatal care can impact health outcomes for premature neonates (29). Studies have identified regional differences in neonatal intensive care unit (NICU) beds, with the Southeast having the most NICUs (26%) and the Southwest having the least (12%). The West had 22% of NICUs (30). Importantly, the Northeast and Southeast had more NICUs with higher acuity level (30).

### Rural vs. urban

Finally, neonatal mortality was analyzed by locality, differentiating urban vs. rural populations. Overall, CMR was higher in the urban group in comparison to the rural group for every year except 2017. This contradicts trends identified in prior studies. For example, in a retrospective cohort of VLBW infants born in California, survival was decreased with increasing rurality (31). In a national study of infant mortality, regardless of prematurity status, infant mortality was higher in rural areas (32). However, in this study, CMR did significantly decrease in the urban population while it did not in the rural population. One potential explanation for lack of improvement in rural neonatal mortality includes access to higher level NICU centers which are concentrated in urban areas (30). In some regions, this may be associated with decreased survival in rural areas in comparison to urban areas. Antenatal interventions, like antenatal corticosteroid prophylaxis, are important to survival of extremely immature neonates. It is well known that rural areas lack access to obstetric care, with many rural counties in the US completely without access or losing access to obstetric care (33). Thus, it would be expected that rural areas would have higher neonatal mortality in comparison urban areas. However, continued loss of access to obstetric care likely contributes to lack of improvement in rural neonatal mortality over the past two decades.

The difference in mortality between rural and urban areas cannot be fully explained by differences in preterm birth rates. In a national retrospective cohort analysis of premature births, researchers identified a premature birth rate of 10.02% in urban areas and a rate of 10.19% in rural areas (34). Though this study included all premature births less than 37 weeks in the analysis, the preterm birth rates are near identical between rural and urban populations.

Still, the trends in urban vs. rural mortality contradict prior literature. Potential biases may influence this data. For example, there may be complicated cases that are referred to urban centers with higher levels of care prior to birth. Additionally, rural neonates may be transferred to tertiary urban centers after delivery. Deaths in these neonates may be reported as urban deaths vs. rural deaths, confounding the data. Importantly, data was only stratified by rural vs. urban status through 2020 as rural and urban data was only available on the CDC WONDER database after 2020. Full stratification of the data through 2023 may have highlighted changing trends in the past few years that would better align with prior published literature.

### Impact of COVID-19 pandemic

The study period includes the time of the COVID-19 pandemic. There were no changes in trends at the time of the COVID-19 pandemic when looking at mortality overall and mortality stratified by sex. When looking at mortality stratified by race and ethnicity, there was a trend change in mortality in 2019 for Asian and Pacific Islander neonates signified by a Joinpoint. There were no trend changes in 2019 for other racial or ethnicity groups. There no trend changes at the time of the COVID-19 pandemic for different regions. Data was only stratified for urban vs. rural localities until 2020 due to changes in CDC WONDER reporting. Therefore, it is difficult to assess for trend change at the time of the COVID-19 pandemic in urban vs. rural sub-populations. Overall, it does not appear that the COVID-19 pandemic was a time of significant change in neonatal mortality rates. Interestingly, the first few months of lockdown has been associated with a decrease in preterm birth rates worldwide (35). In a study of preterm birth rates in two US cities during the pandemic, no significant change in preterm birth rate was identified (36). A study of neonates in Alabama identified an increase in moderately preterm births but no change in neonatal mortality across the pandemic period (37). While those studies may not have been representative of the national population as a whole, they do align with the data in this study.

## Conclusion

This study sought to determine trends in neonatal mortality associated with extreme prematurity from 1999 to 2023 in the US. Special attention was paid to disparities in neonatal mortality rates during this time period. While CMR decreased overall across the study period, significant disparities were identified. NH Black neonatal mortality was higher than any other racial or ethnic group. Mortality failed to significantly decrease in Hispanic and NH Asian American neonates. Mortality did decrease in all regions except for the West. While mortality was higher in urban areas than rural areas, it did decrease in urban areas while it did not in rural areas. Despite medical advances in antenatal and neonatal interventions, certain groups still experience higher burden of mortality in the US. This study highlights clear areas for improvement in the US to improve neonatal mortality among extremely premature neonates. Future research should explore maternal and perinatal risk factors, confounders, and underlying causes of death to better target interventions. Importantly, this study demonstrates an overall improvement in mortality, but further studies are required to determine trends in morbidity as well as associated disparities.

## Limitations

This CDC WONDER database study, based on death certificate data, is limited by the lack of detailed clinical information, including specific causes of death and whether resuscitation was attempted. The inability to stratify by narrower age ranges also restricts analysis of mortality trends across infancy. Additionally, underreporting or misclassification on death certificates may lead to missed cases or inclusion of inappropriate cases. Further, it is not possible to link deaths to specific maternal or perinatal risk factors that may have led to preterm birth. There are multiple confounders that are not included in the data that can influence neonatal mortality including but not limited to congenital malformations, genetic conditions, infection, and events during birth. This limits the ability to determine the exact underlying cause of disparities in mortality in extremely premature infants. Other potential confounders not accounted for in this aggregated data set include birth weight, sex ratio, socioeconomic gradients, and regional NICU distribution among others. Future studies including such confounders are needed to better elucidate underlying cause of disparities in neonatal mortality. Importantly, mortality trends among American Indian infants are difficult to assess due to low case numbers. Further, rural vs. urban locality data was only available on the CDC WONDER database through 2020. This limits the ability to assess recent trends in neonatal mortality in rural vs. urban areas. Despite these limitations, the study offers valuable insight into national mortality trends in extremely premature infants from 1999 to 2023.

## Data Availability

The original contributions presented in the study are included in the article/Supplementary Material, further inquiries can be directed to the corresponding author.
