# Supplementary material for: Mortality trends in extremely premature neonates: insights from the CDC WONDER database from 1999 to 2023
Source: Front Pediatr. 2025 Nov 20;13:1683346. doi: 10.3389/fped.2025.1683346 (PMC12675377; doi:10.3389/fped.2025.1683346)
Supplement: Supplementary file 1 [file Supplementaryfile1.docx]

Supplementary Material

# Supplementary Figures and Tables

## Supplementary Tables

|  | Crude Mortality Rate (per 100,000) | | |
| --- | --- | --- | --- |
| Year | Overall | Female | Male |
| 1999 | 93.53 | 85.96 | 100.74 |
| 2000 | 91.63 | 86.66 | 96.36 |
| 2001 | 97.62 | 89.76 | 105.13 |
| 2002 | 106.49 | 96.43 | 116.12 |
| 2003 | 109.91 | 97.14 | 122.12 |
| 2004 | 106.84 | 95.05 | 118.13 |
| 2005 | 108.98 | 96.95 | 120.48 |
| 2006 | 108.94 | 100.36 | 117.14 |
| 2007 | 108.58 | 100.04 | 116.75 |
| 2008 | 107 | 95.85 | 117.69 |
| 2009 | 104.56 | 93.82 | 114.85 |
| 2010 | 98.75 | 90.89 | 106.29 |
| 2011 | 95.76 | 87.41 | 103.73 |
| 2012 | 99.9 | 90.74 | 108.64 |
| 2013 | 96.56 | 89.61 | 103.19 |
| 2014 | 96.5 | 87.08 | 105.51 |
| 2015 | 93.21 | 84.56 | 101.47 |
| 2016 | 92.59 | 81.41 | 103.28 |
| 2017 | 87.22 | 77.23 | 96.77 |
| 2018 | 87.26 | 78.36 | 95.76 |
| 2019 | 83.4 | 73.6 | 92.76 |
| 2020 | 75.07 | 67.44 | 82.38 |
| 2021 | 75.63 | 70.57 | 80.48 |
| 2022 | 69.42 | 63.82 | 74.78 |
| 2023 | 69.89 | 63.67 | 75.84 |
| Number of Joinpoints (Years of Joinpoints) | 2 (2003, 2016) | 2 (2006, 2015) | 2 (2003, 2017) |
| APC Segment 1 (95% CI) | 5.46* (3.33, 8.89) | 2.05* (0.96, 3.76) | 6.31* (3.68, 11.54) |
| APC Segment 2 (95% CI) | -1.38* (-1.79, -0.89) | -1.87 (-2.48, 1.13) | -1.47* (-1.95, -0.92) |
| APC Segment 3 (95% CI) | -4.30* (-6.00, -3.35) | -3.58* (-6.14, -2.75) | -4.93* (-8.27, -3.56) |
| Average APC (95% CI) | -1.14* (-1.45, -0.83) | -1.32* (-1.63, -1.06) | -1.10* (-1.57, -0.68) |

**Supplementary Table 1.** Extreme Prematurity Related Crude Mortality Data, Overall and Stratified by Sex, 1999-2023.

|  | Crude Mortality Rate (per 100,000) | | | | |
| --- | --- | --- | --- | --- | --- |
| Year | American Indian | Asian or Pacific Islander | Black or African American | White | Hispanic |
| 1999 | 66.21 | 42.65 | 259.18 | 60.13 | 72.51 |
| 2000 | 66.21 | 53.39 | 255 | 58.31 | 65.75 |
| 2001 | 66.21 | 41.01 | 258.98 | 68.83 | 68.71 |
| 2002 | 66.21 | 63.89 | 278.06 | 73.96 | 79 |
| 2003 | 66.21 | 62.31 | 283.47 | 75.83 | 89 |
| 2004 | 66.21 | 65.26 | 273.08 | 75.64 | 83.94 |
| 2005 | 66.21 | 68.44 | 277.98 | 75.55 | 87.81 |
| 2006 | 66.21 | 62.03 | 284.77 | 74.64 | 85.72 |
| 2007 | 66.21 | 72.91 | 273.65 | 77.13 | 81.14 |
| 2008 | 66.21 | 74.18 | 261.01 | 73.55 | 87.51 |
| 2009 | 66.21 | 58.61 | 263.3 | 72.29 | 80.33 |
| 2010 | 66.21 | 70.8 | 235.2 | 68.13 | 81.29 |
| 2011 | 66.21 | 64.7 | 231.32 | 68.72 | 71.37 |
| 2012 | 66.21 | 59.57 | 238.75 | 71.82 | 75.36 |
| 2013 | 66.21 | 60.69 | 224.09 | 70.31 | 74.88 |
| 2014 | 66.21 | 63.65 | 228.42 | 68.63 | 74.91 |
| 2015 | 66.21 | 60 | 218.38 | 64.53 | 77.53 |
| 2016 | 66.21 | 72.17 | 224.37 | 63.12 | 73.93 |
| 2017 | 66.21 | 66.23 | 211.72 | 57.17 | 72.95 |
| 2018 | 66.21 | 74.01 | 211.24 | 58.26 | 68.68 |
| 2019 | 66.21 | 78.24 | 203.78 | 53.48 | 69.55 |
| 2020 | 66.21 | 40.49 | 176.83 | 52.02 | 62.14 |
| 2021 | Unreliable | 57.63 | 184.22 | 50.23 | 68.64 |
| 2022 | Unreliable | 48.58 | 158.16 | 47.55 | 62.02 |
| 2023 | Unreliable | 43.26 | 164.47 | 42.69 | 69.34 |
| Number of Joinpoints (Years of Joinpoints) | 1 (2002) | 1 (2019) | 2 (2005, 2018) | 2 (2003, 2014) | 1 (2004) |
| APC Segment 1 (95% CI) | 0 (0,0) | 1.03* (0.059, 2.89) | 1.55 (-2.57, 6.93) | 7.25* (4.98, 10.76) | 5.57* (2.44, 11.55) |
| APC Segment 2 (95% CI) | 0* (0,0) | -12.49* (-32.10, -3.31) | -2.3773 (-2.99, 5.12) | -1.0897* (-1.65, -0.44) | -1.58* (-2.09,1.17) |
| APC Segment 3 (95% CI) | - | - | -5.0466*(-11.91, -2.77) | -4.66* (-5.79, -3.92) | - |
| Average APC (95% CI) | 0.00* (0,0) | -1.36 (-2.99, 0.051) | -1.98* (-2.62, -1.46) | -1.12* (-1.42,-0.82) | -0.13 (-0.57,0.42) |

**Supplementary Table 2.** Extreme Prematurity Related Crude Mortality Data Stratified by Race and Ethnicity, 1999-2023.

|  | Crude Mortality Rate (per 100,000) | | | |
| --- | --- | --- | --- | --- |
| Year | Northeast | Midwest | South | West |
| 1999 | 82.52 | 106.59 | 108.25 | 66.84 |
| 2000 | 81.22 | 111.68 | 104.35 | 60.79 |
| 2001 | 93.32 | 112.39 | 110.32 | 67.85 |
| 2002 | 104.14 | 114.92 | 123.03 | 75.46 |
| 2003 | 112.84 | 121.93 | 121.31 | 80.11 |
| 2004 | 99.19 | 114.38 | 126.07 | 76.22 |
| 2005 | 104.55 | 122.19 | 120.48 | 82.97 |
| 2006 | 111.15 | 122.09 | 121.24 | 77.39 |
| 2007 | 108.44 | 124.82 | 123.73 | 71.38 |
| 2008 | 112.07 | 113.57 | 122.35 | 74.46 |
| 2009 | 107.46 | 120.12 | 114.79 | 73.22 |
| 2010 | 104.13 | 118.56 | 105.82 | 67.16 |
| 2011 | 99.51 | 108.56 | 106.31 | 65.89 |
| 2012 | 109.88 | 118.62 | 108.64 | 64.32 |
| 2013 | 92.44 | 111.6 | 105.82 | 72.08 |
| 2014 | 101.87 | 109.55 | 106.64 | 66.19 |
| 2015 | 94.13 | 102.25 | 104.42 | 67.34 |
| 2016 | 84.1 | 111.22 | 107.32 | 59.11 |
| 2017 | 78.11 | 117.26 | 93.29 | 57.91 |
| 2018 | 80.6 | 104.83 | 94.8 | 64.67 |
| 2019 | 69.24 | 98.46 | 98.22 | 55.85 |
| 2020 | 60.67 | 89.25 | 86.32 | 53.78 |
| 2021 | 65.04 | 89.28 | 83.02 | 58.45 |
| 2022 | 48.46 | 84.17 | 79.15 | 54.3 |
| 2023 | 50.49 | 85.46 | 80.38 | 51.1 |
| Number of Joinpoints (Years of Joinpoints) | 2 (2003, 2014) | 2 (2005, 2017) | 2 (2004, 2016) | 1 (2004) |
| APC Segment 1 (95% CI) | 8.40* (4.38, 17.63) | 2.05* (0.52, 6.74) | 3.59* (0.72, 9.17) | 4.90* (1.12, 14.30) |
| APC Segment 2 (95% CI) | -0.73 (-1.97, 0.39) | -0.98* (-1.93, -0.21) | -1.73 (-2.35, 3.76) | -2.15* (-3.01, -1.64) |
| APC Segment 3 (95% CI) | -7.47* (-9.70, -6.09) | -4.65* (-8.71, -3.10) | -3.64* (-9.37, -2.32) | - |
| Average APC (95% CI) | -1.89* (-2.50, -1.26) | -1.17* (-1.60, -0.77) | -1.21* (-1.71, -0.74) | -0.72 (-1.32, 0.19) |

**Supplementary Table 3.** Extreme Prematurity Related Crude Mortality Data Stratified by Region, 1999-2023.

|  | Crude Mortality Rate (per 100,000) | |
| --- | --- | --- |
| Year | Rural | Urban |
| 1999 | 82.56 | 95.38 |
| 2000 | 73.99 | 94.63 |
| 2001 | 83.07 | 100 |
| 2002 | 88.52 | 109.43 |
| 2003 | 95.67 | 112.24 |
| 2004 | 90.81 | 109.49 |
| 2005 | 97.16 | 110.95 |
| 2006 | 88 | 112.44 |
| 2007 | 88.45 | 111.98 |
| 2008 | 91.92 | 109.56 |
| 2009 | 89.65 | 107.06 |
| 2010 | 82.92 | 101.36 |
| 2011 | 82.65 | 97.84 |
| 2012 | 92.77 | 101.02 |
| 2013 | 86.01 | 98.2 |
| 2014 | 87.19 | 97.95 |
| 2015 | 84.97 | 94.5 |
| 2016 | 80.23 | 94.49 |
| 2017 | 88.27 | 87.06 |
| 2018 | 76.64 | 88.89 |
| 2019 | 79.49 | 84 |
| 2020 | 71.12 | 75.69 |
| Number of Joinpoints (Years of Joinpoints) | 1 | 3 |
| APC Segment 1 (95% CI) | 5.31* (1.00, 15.49) | 4.63* (2.53, 8.22) |
| APC Segment 2 (95% CI) | -1.06* (-2.04, -0.56) | -0.17 (-2.52, 3.35) |
| APC Segment 3 (95% CI) | - | -2.02* (-2.55, -0.68) |
| APC Segment 4 (95% CI) | - | -7.02* (-9.90, -3.43) |
| Average APC (95% CI) | 0.12 (-0.51, 0.96) | -0.93* (-1.24, -0.59) |

**Supplementary Table 4.** Extreme Prematurity Related Crude Mortality Data Stratified by Rural versus Urban Locale, 1999-2023.

## Supplementary Figures


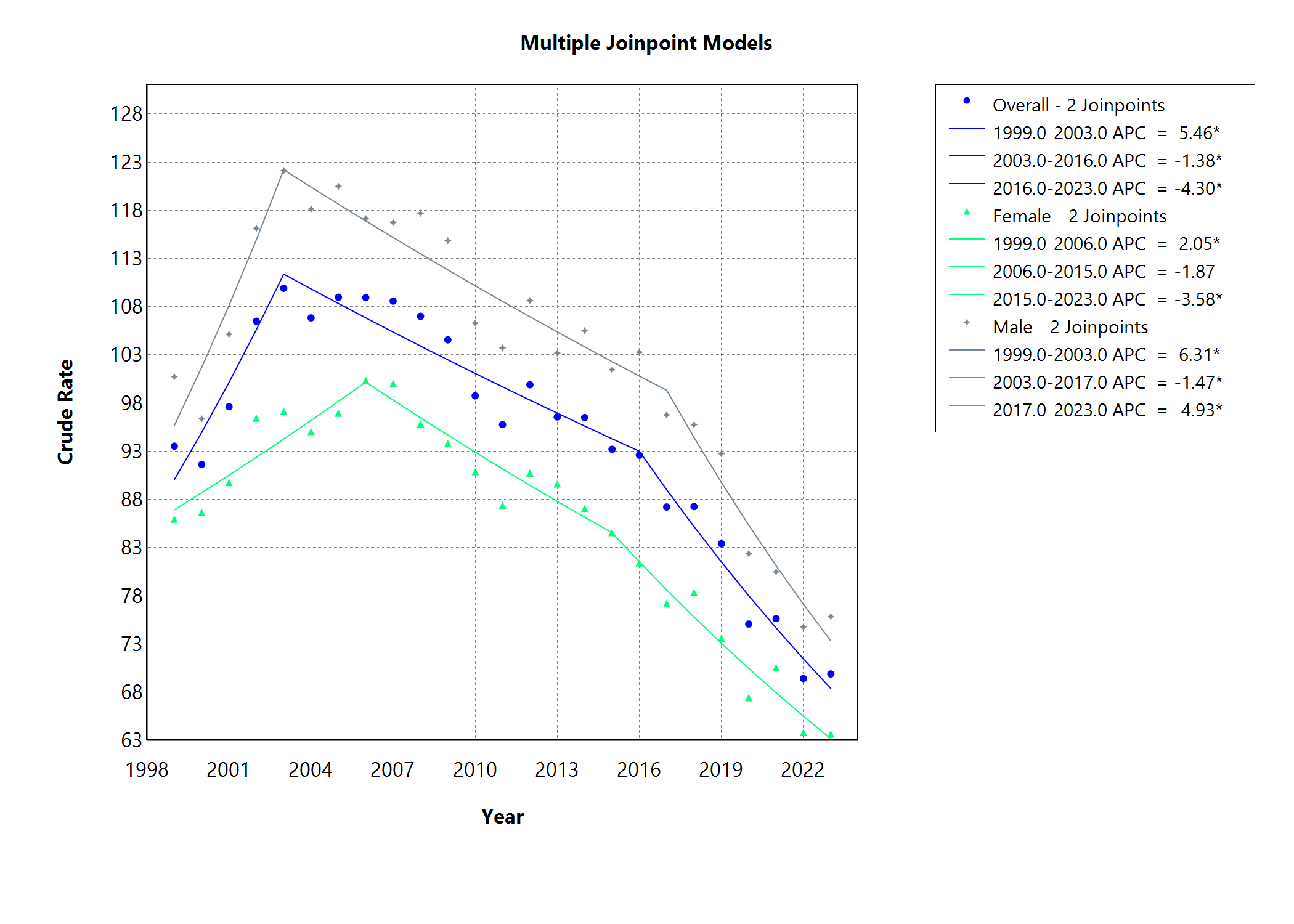
**Supplemental Figure 1.** Multiple Joinpoint Model of Extreme Prematurity Related Deaths Stratified by Sex, 1999-2023.


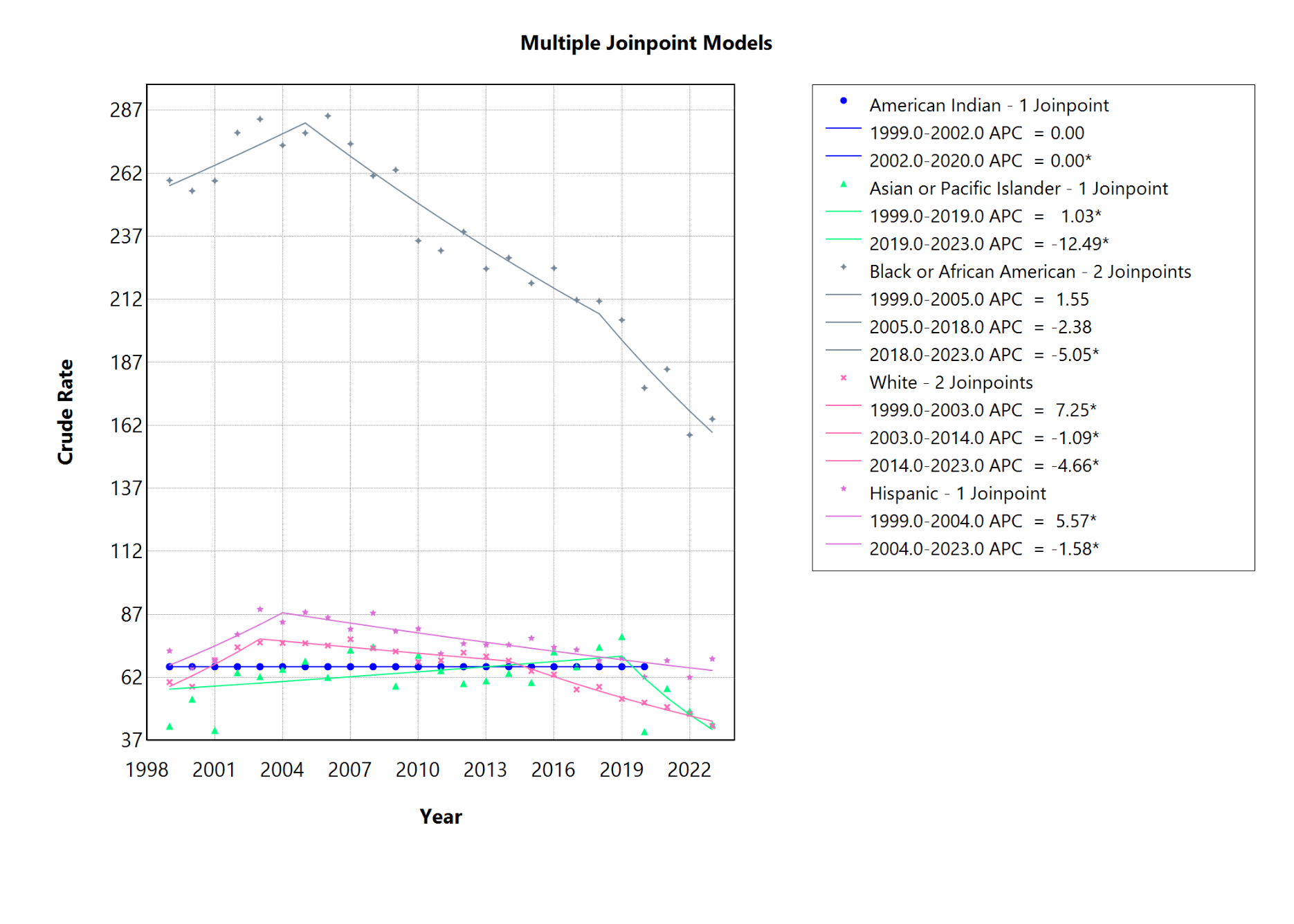
**Supplemental Figure 2.** Multiple Joinpoint Model of Extreme Prematurity Related Deaths Stratified by Race and Ethnicity, 1999-2023.


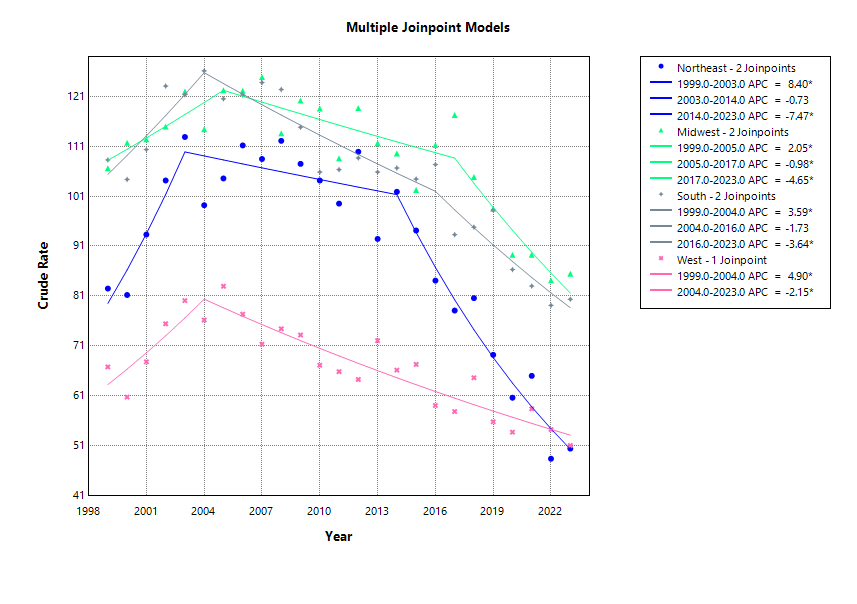
**Supplemental Figure 3.** Multiple Joinpoint Model of Extreme Prematurity Related Deaths Stratified by Region, 1999-2023.


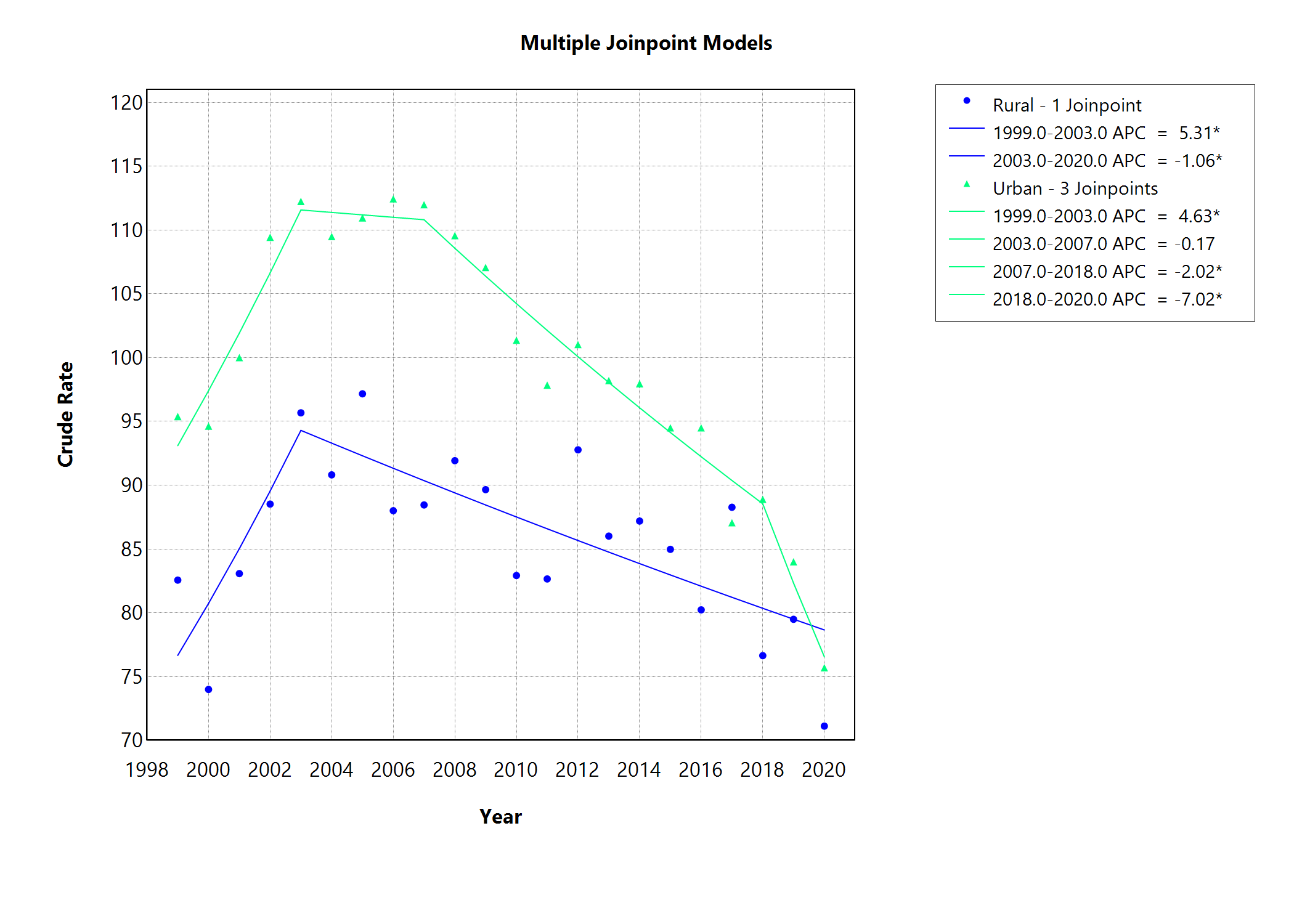
**Supplemental Figure 4.** Multiple Joinpoint Model of Extreme Prematurity Related Deaths Stratified by Rural versus Urban Locality, 1999-2020.
